# Individual structural differences in left inferior parietal area are associated with schoolchildrens' arithmetic scores

**DOI:** 10.3389/fnhum.2013.00844

**Published:** 2013-12-06

**Authors:** Yongxin Li, Yuzheng Hu, Yunqi Wang, Jian Weng, Feiyan Chen

**Affiliations:** ^1^Bio-X Laboratory, Department of Physics, Zhejiang UniversityHangzhou, China; ^2^School of International Studies, Zhejiang UniversityHangzhou, China

**Keywords:** arithmetical skill, left intraparietal sulcus, structure-behavior correlation, voxel-based morphometry, fiber tracking

## Abstract

Arithmetic skill is of critical importance for academic achievement, professional success and everyday life, and childhood is the key period to acquire this skill. Neuroimaging studies have identified that left parietal regions are a key neural substrate for representing arithmetic skill. Although the relationship between functional brain activity in left parietal regions and arithmetic skill has been studied in detail, it remains unclear about the relationship between arithmetic achievement and structural properties in left inferior parietal area in schoolchildren. The current study employed a combination of voxel-based morphometry (VBM) for high-resolution T1-weighted images and fiber tracking on diffusion tensor imaging (DTI) to examine the relationship between structural properties in the inferior parietal area and arithmetic achievement in 10-year-old schoolchildren. VBM of the T1-weighted images revealed that individual differences in arithmetic scores were significantly and positively correlated with the gray matter (GM) volume in the left intraparietal sulcus (IPS). Fiber tracking analysis revealed that the forceps major, left superior longitudinal fasciculus (SLF), bilateral inferior longitudinal fasciculus (ILF) and inferior fronto-occipital fasciculus (IFOF) were the primary pathways connecting the left IPS with other brain areas. Furthermore, the regression analysis of the probabilistic pathways revealed a significant and positive correlation between the fractional anisotropy (FA) values in the left SLF, ILF and bilateral IFOF and arithmetic scores. The brain structure-behavior correlation analyses indicated that the GM volumes in the left IPS and the FA values in the tract pathways connecting left IPS were both related to children's arithmetic achievement. The present findings provide evidence that individual structural differences in the left IPS are associated with arithmetic scores in schoolchildren.

## Introduction

Arithmetic skill is of critical importance for academic achievement (Duncan et al., 2007), professional success (Gross et al., 2009) and everyday life. This skill, one of the many fascinating abilities that humans are endowed with, is generally gained though life experience and school instruction. Childhood is the key period for individuals to learn how to process abstract numerical symbols and perform mental arithmetic (Ansari, 2008). Children's arithmetic skill was found to develop cumulatively in the elementary school, and inter-individual differences in arithmetic skill increased over time (Kikas et al., 2009). The investigation of the neural correlates associated with mathematical cognition in schoolchildren would shed some light on school education.

Recent brain imaging research also has advanced our understanding of the cerebral networks involved in the acquisition of arithmetic skill (Dehaene et al., 2004; Rivera et al., 2005; Arsalidou and Taylor, 2011). The fronto-parietal network (especially the inferior parietal area) has been consistently observed to be activated in arithmetic processing in both adults and children (Dehaene et al., 2003; Venkatraman et al., 2005; Ischebeck et al., 2007; Price et al., 2013). Within the left inferior parietal area, the intraparietal sulcus (IPS) is assumed to be essential for the appreciation of quantity (Dehaene et al., 2004). The recruitment of the left inferior parietal cortex during calculation was found to increase with age in the population aged between 8 and 19 years old (Rivera et al., 2005). Similarly, Bugden et al. (2012) found that children with more mature response modulation on the left IPS during symbolic number processing exhibited higher arithmetic competence. The left IPS was consistently identified as a key neural substrate for number and arithmetic operations in previous functional imaging studies (Dehaene et al., 2003, 2004; Rivera et al., 2005; Price et al., 2013). Furthermore, findings of brain structural imaging studies also support this viewpoint. For example, a recent study showed that children with low numerical proficiency had less gray matter (GM) volume in the parietal areas (particularly in the left IPS and bilateral angular gyri) than those with high numerical proficiency (Lubin et al., 2013). Aydin et al. (2007) found that cortical GM density in the bilateral inferior parietal lobule was significantly higher in adult mathematicians than in the controls. Studies using diffusion tensor imaging (DTI) also revealed significant and positive correlation between arithmetical scores and fractional anisotropy (FA) values in white matter (WM) tracts linking the left inferior parietal area to other brain areas in both healthy subjects (van Eimeren et al., 2008; Tsang et al., 2009) and abnormal subjects with arithmetic dysfunction (Barnea-Goraly et al., 2005; Rykhlevskaia et al., 2009; Lebel et al., 2010).

The above described findings indicate that inter-individual differences in arithmetic ability have a link to cerebral activation, GM volume/density and WM integrity in the inferior parietal area respectively. And inter-individual differences in arithmetic skill of schoolchildren increase over time. Childhood is the key period for individuals to gain mathematic knowledge. However, it remains unclear about the relationship between arithmetic achievement and structural properties in left inferior parietal area in schoolchildren. Addressing this question should contribute to our understanding of the neural mechanisms that underlie mathematical cognition and shed some light on school education. As the inferior parietal area had been suggested to play a critical role in arithmetic processing (Rivera et al., 2005; van Eimeren et al., 2008; Bugden et al., 2012; Lubin et al., 2013; Price et al., 2013), the present study was designed to examine the relationship between structural properties of this region and arithmetic achievement in schoolchildren about 10 years old. We tested children in this age range for the following two reasons. Firstly, childhood is the key time period when humans learn how to process abstract numerical symbols and perform mental arithmetic (Ansari, 2008). Secondly, at this particular range, children typically attend 4th graders, by which they have been formally exposed to decimal system and equipped with basic arithmetic skills such as addition, subtraction, multiplication and division. They typically demonstrate remarkable general arithmetical ability but at the same time substantial individual differences. Therefore, we think children within this age range constitute a suitable population for examining the neural substrate of arithmetic ability.

Based on the previous studies, we hypothesized that individual differences in schoolchildrens' arithmetic skill would be correlated with brain structural variability in the GM volumes of the left IPS and integrity of WM tracts linking to this region. To test our hypothesis, voxel-based morphometry (VBM) for T1-weighted images and fiber tracking on DTI images were used to examine children's brain structures and the arithmetic subtest of the Chinese-Revised Wechsler Intelligence Scale for Children (WISC-RC) was employed to assess their arithmetic ability.

## Materials and methods

### Subjects

Fifty-nine 4th graders attended the present experiment (mean age ± *SD*, 10.48 ± 0.41 years; age range: 9.64–11.33 years; 28 girls). All children were recruited from Beiguang Primary School of Weifang, China, and all were from urban families. None of the participants reported history of a neurological or psychiatric disorder. Informed written consent was obtained from both children and their parents before the MRI scan. This study was approved by Zhejiang University.

### Cognitive assessment

The WISC-RC was administered during the week of MRI data collection to assess the intelligence of children (mean IQ ± *SD*, 110.42 ± 9.89; range = 85–130). The arithmetic achievement was measured with the WISC-RC arithmetic subtest. The arithmetic subtest of the WISC-RC, including 15 questions, measures numerical reasoning and the ability to solve arithmetic problems (Sattler, 2001; Barnea-Goraly et al., 2005). The participants were instructed to mentally answer these questions without using pen and paper. Arithmetic scores for all children ranged between 8 and 15 (mean score ± *SD*, 11.59 ± 1.81).

### MRI data acquisition

The MRI data were acquired on a 3T Philips MR scanner with an eight-channel Philips SENSE head coil. Whole-brain anatomical images were obtained by using a T1-weighted three-dimensional gradient echo pulse sequence. The following acquisition parameters were used: repetition time, 7 ms; echo time, 2.2 ms; flip angle, 25; field-of-view, 230 × 230 mm^2^; voxel size, 0.9 × 0.9 × 1 mm^3^, 164 sagittal slices. The DTI data was collected using single-shot echo planar imaging sequence (repetition time, 14000 ms; echo time, 70.2 ms; field-of-view, 230 × 230 mm^2^; the acquisition matrix, 256 × 256, 66 slices; slice thickness, 2 mm). Diffusion gradient was applied along 30 directions (*b* = 800 s/mm^2^) with one volume without diffusion weighting (*b* = 0 s/mm^2^).

### MR data analysis

#### VBM analysis of the anatomical MRI image

VBM for T1-weighted image has been widely used to examine the structural changes within the whole brain without bias and to find significant regional differences by applying voxel-wise statistics in the context of Gaussian random fields (Ashburner and Friston, 2000). T1-weighted high-resolution data were analyzed using the VBM8 toolbox in the SPM8 software package (Welcome Department of Imaging Neuroscience Group, London, UK) running on MATLAB platform (version 7.11, Mathworks, Natick, MA). Non-linear normalization was achieved by diffeomorphic anatomical registration through exponentiated lie algebra toolbox (DARTEL) analysis (Ashburner, 2007).

In the first step, each T1-weighted structural scan was spatially normalized into stereotactic space by coregistering with the standard Montreal Neurological Institute (MNI152) brain template. The coregistered images were segmented to obtain a bias-corrected structural image that has more uniform intensities within different tissue classes, including GM, WM, and cerebrospinal fluid (CSF), and produced separate GM, WM, and CSF images (Ashburner and Friston, 2005). To ensure data quality, segmented images were checked via the module of “check data quality” in the VBM8 toolbox (http://dbm.neuro.uni-jena.de/vbm/). Volumes with an overall covariance below two standard deviations would be indicated as poor quality and be discarded. None of the subjects was excluded due to poor data quality. In the second step, a customized, more population-specific template was created using the DARTEL (Ashburner, 2007). Each subject's GM map was transformed to the customized template space and then to the MNI standard space. The warped GM images were modulated by the Jacobian determinants derived from the spatial normalization step to obtain the GM volume (Good et al., 2001). The modulated GM images were written with an isotropic voxel resolution of 1.5 × 1.5 × 1.5 mm^3^. Finally, these images were smoothed with a full width at half maximum kernel of 6 mm to improve the normality of the data distribution and reduce the number of false positives. Additionally, an absolute threshold mask of 0.1 was used to avoid any possible overlapping edge effect between the GM and WM.

SPM8 was used for all statistical analyses. A multiple linear regression model was used to analyze the correlation between the local GM volumes and scores on WISC-RC arithmetic subtest, on a voxel-by-voxel basis. Full-scale IQ, age and gender were added as confounding covariates in this analysis. Additionally, we used the bilateral IPS masks from the automated anatomical labeling template as an explicit mask to restrict the analysis in the inferior parietal regions (Tzourio-Mazoyer et al., 2002). The statistical threshold was set to *p* < 0.05 using the AlphaSim correction (with a threshold of *p* < 0.01 and a minimum cluster size of 76 voxels). This correction was made using the AlphaSim program in the REST software (http://restfmri.net/forum/rest) with the parameter FWHM = 6 mm. This program applied the Monte Carlo simulation using both the individual voxel probability threshold and the cluster size to calculate the probability of detecting a false positive (Song et al., 2011). Wherever a significant correlation was found between regional GM volume and arithmetic scores, the GM volumes were extracted from the significant cluster(s) into SPSS, and regression line between the GM volumes in the left IPS and the WISC-RC arithmetic scores for entire cohort was drawn. All the correlation analyses were adjusted for full-scale IQ, age and gender.

#### DTI: probabilistic tractography analysis

DTI is an advanced imaging technique that can be used to examine the integrity of the neural pathways, an index of WM health at the microstructure level. This technique provided useful information to characterize microstructural properties of the brain tissue, such as axonal fiber density, axonal diameter, and degree of myelination (Johansen-Berg and Behrens, 2009; Zatorre et al., 2012). Single-subject and group-level DTI analyses were performed using the FMRIB Software Library (FSL v4.1.9, www.fmrib.ox.ac.uk/fsl). First, the raw diffusion data were corrected for local field distortions and head movements using an affine registration to the first non-diffusion-weighted image. Next, the non-brain tissue and background noise were removed using the Brain Extraction Tool (BET v2.1) (Smith, 2002). Finally, the FMRIB's Diffusion Toolbox (FDT v2.0) was used to fit the diffusion tensor and calculate the FA, eigenvector and eigenvalue maps. All DTI images were inspected visually and discarded if judged to be of poor quality (e.g., in the V1 color maps, the color of known WM tracts don't correspond to the known axonal orientation). The DTI data for twelve subjects (5 girls) were excluded due to motion artifacts. The tract-based spatial statistics (TBSS) processing pipeline was used to align the main fiber tracts to a standard brain space. The TBSS procedure was described in detail elsewhere (Smith et al., 2007). Briefly, FA images of all subjects were first aligned into a standard brain space using non-linear registration. Next, the mean of all subjects' aligned FA images was created, and then ‘thinned’ using a projection technique to create a mean FA skeleton that represents the centers of major tracts common to all subjects. Each subject's aligned FA data was then projected onto this skeleton. This projection data were used in the following analysis to extract the mean FA values.

The tractography analysis was carried out using the program PROBTRACKX (Probabilistic Tractography of Crossing fibers) (Behrens et al., 2003b, 2007). The regions showed significant correlation between local GM volume and arithmetic scores were selected as the seed masks to delineate the pathways. The seed masks were linearly transformed into the native space of each subject. The standard parameters were used: 5000 sample tracts per seed voxel, a curvature threshold of 0.2, a step length of 0.5, and the maximum number of steps set at 2000. Fiber tracking resulted in a probabilistic map of the connections of the voxels included in the starting seed with the rest of the brain. Each voxel in the tract map had an intensity value that represented the number of tractography runs that were successfully able to pass in that voxel. To remove the spurious connections, we excluded the voxels through which no more than 50 samples passed (out of the 5000 streamline samples from each seed voxel) from the following analyses. These selected pathways from each subject were then binarized, transformed to standard space, and summed across the subjects to produce probability maps. The probabilistic map was set at a threshold at which the paths were present in at least of one-third of subjects (Song et al., 2012). The John Hopkins University (JHU) WM tractography atlas was used for tract labeling (Mori et al., 2008).

Finally, all probabilistic maps were combined to generate a mask that was used in the following statistical analysis. Using this mask, we aimed to examine the correlation between FA values and arithmetic scores. The FA images from all of the subjects were first realigned to a common target and then normalized to a 1 × 1 × 1 mm^3^ MNI152 standard space via the FMRIB58_FA template. Each subject's aligned FA data were smoothed to a Gaussian kernel of 6 mm to minimize individual variability, improve the normality of the data distribution and reduce the number of false positives. The smoothed FA maps were then subjected to a multiple linear regression analysis, in which regional WM FA values were used as a dependence variable, and full-scale IQ, age, gender were used as independent variables to investigate the correlation between regional WM FA values and arithmetic scores. Clusters were considered as significant at the combined voxel-extend threshold of an uncorrected voxel level of *p* < 0.01 and cluster extent > 473 voxels, as determined based on Monte Carlo simulation with AlphaSim correlation to *p* < 0.05. The JHU WM tractography atlas was used to determine the locations of the significant voxels in the voxel-based correlation analysis. A *post-hoc* confirmatory analysis was conducted using the cluster that showed significant correlation between FA values and arithmetic scores as regions of interest. The mean FA values within this region of interest were extracted from each subject's WM skeleton and exported to SPSS. Regression line between the mean FA values in the WM clusters and the WISC-RC arithmetic scores for entire cohort was drawn. All the correlation analyses above were adjusted for full-scale IQ, age and gender.

## Results

### Gray matter volume

**Figure 1:**
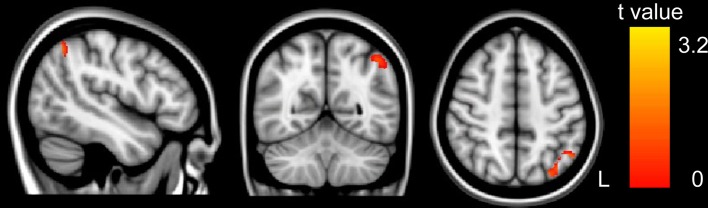
**The correlation between GM volume and WISC-RC arithmetic scores**. Positive correlation between GM volume and WISC-RC arithmetic scores in the left IPS. The location of the significantly correlated cluster was shown in red-yellow. The color intensity represents the T-statistical value at the voxel level. The statistical threshold was set at *p* < 0.05 using the AlphaSim correction (with a threshold of *p* < 0.01 and a minimum cluster size of 76 voxels). L, left. There were no negative correlations between GM volume and arithmetic score.

**Figure 2:**
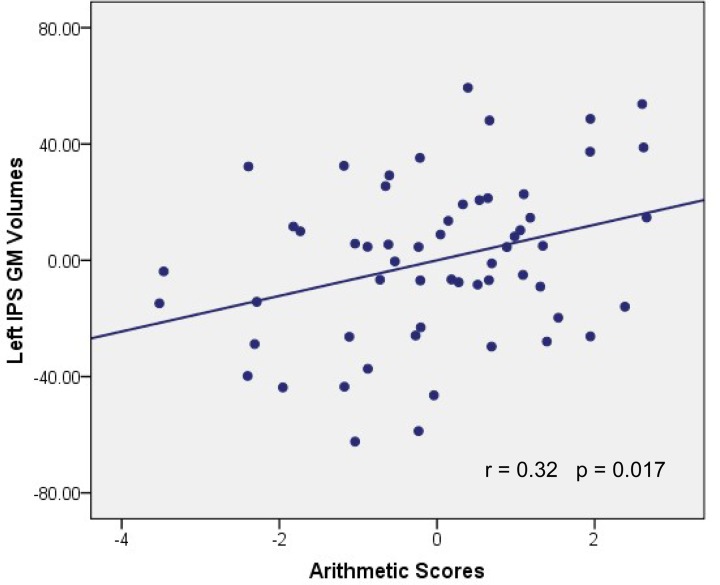
**Association between GM volume and WISC-RC arithmetic scores**. Parietal residual plot with trend lines depicting the correlation between residuals in multiple regression analysis investigating the association between the GM volume in the left IPS and the WISC-RC arithmetic scores for entire cohort, after correction of full-scale IQ, age and gender. r denotes the partial correlation coefficient.

GM volumes of the left IPS were found to be significantly and positively correlated with arithmetic scores after adjusting for full-scale IQ, age and gender (MNI coordinate: *x* = −53, *y* = −61, *z* = 43, *t* = 2.38, *p* < 0.01, number of voxels: 381), as shown in Figure 1. Furthermore, the cluster showing significantly correlated with arithmetic scores was extracted as GM mask. The sum of the voxel GM volumes inside this mask was calculated for each subject and exported to SPSS for analysis. The regression line between the GM volumes and the arithmetic scores was drawn (see Figure 2; Table 2).

### WM structure and microstructure

**Figure 3:**
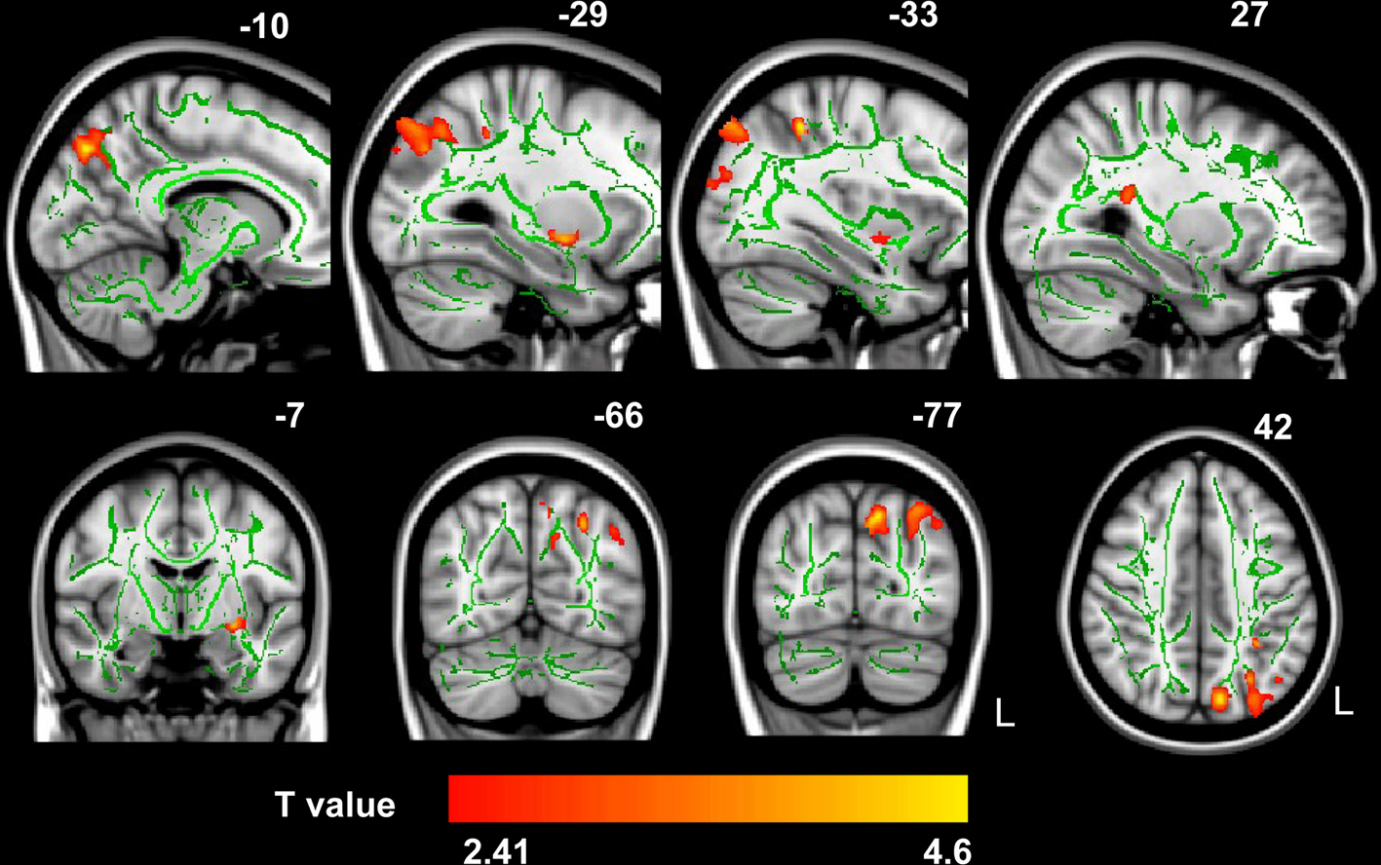
**The correlation between FA values and WISC-RC arithmetic scores**. Positive correlation between FA values and WISC-RC arithmetic scores in the left SLF, ILF, and bilateral IFOF. The location of the significantly correlated cluster was shown in red-yellow. The color intensity represents the T-statistical value at the voxel level. The statistical threshold was set at *p* < 0.05 using the AlphaSim correction (with a threshold of *p* < 0.01 and a minimum cluster size of 473 voxels). The statistical map was superimposed onto the mean of FA skeleton (green) and MNI152 1 × 1 × 1 mm^3^ template (gray-scale) for visualization purpose. L, left. There were no negative correlations between mean FA values and arithmetic scores.

**Figure 4:**
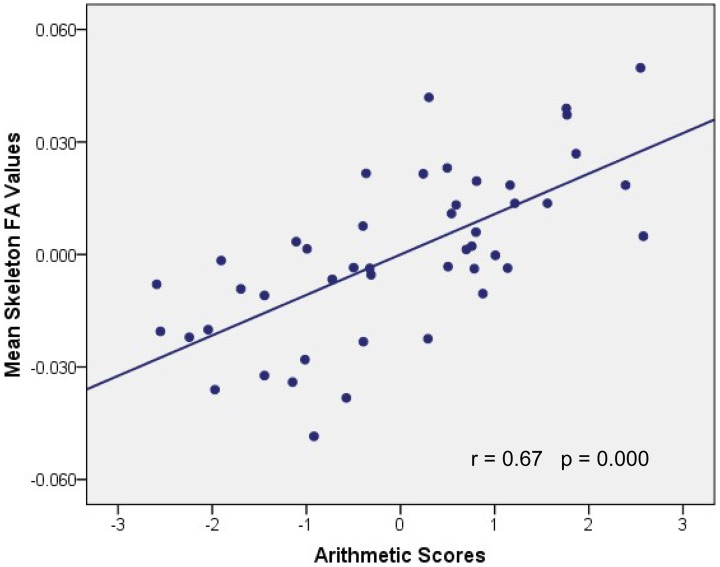
**Association between mean FA values and WISC-RC arithmetic scores**. Parietal residual plot with trend lines depicting the correlation between residuals in multiple regression analysis investigating the association between the mean FA values in the WM clusters and the WISC-RC arithmetic scores for entire cohort, after correction of full-scale IQ, age, and gender. *r* denotes the partial correlation coefficient.

**Figure A1:**
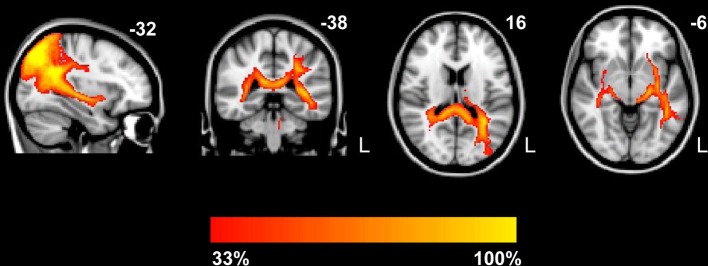
**Probabilistic maps of the tracts**. Group probabilistic maps were set at a threshold so that only the paths present in at least one-third of the subjects are displayed. The fiber tracts include the forceps major, left SLF, bilateral ILF and inferior fronto-occipital fasciculus (IFOF). The color scale indicates the degree of overlap among subjects. L, left.

Probabilistic tractography provides a probability distribution of fiber pathways between seed and target voxles (Behrens et al., 2003a). The probabilistic tracking results indicated that the fiber pathways connecting left IPS with other brain areas included the forceps major, left SLF, bilateral ILF and inferior fronto-occipital fasciculus (IFOF) (see Figure A1 in the Appendix). Voxel-based regression analysis revealed that FA values in the left SLF, ILF and bilateral IFOF showed a significant and positive correlation with arithmetic scores (see Figure 3; Table 1). Furthermore, mean FA values were extracted from these WM clusters and exported to SPSS for analysis. The regression line between WM FA values and arithmetic scores was drawn (see Figure 4; Table 2).

**Table 1:** **Location and MNI coordinates were given for the clusters with significant correlation between fractional anisotropy and WISC-RC math scores**.

**Statistical values**		**MNI coordinates anatomical location**			
**Cluster size**	***t*-value**	***x***	***y***	***z***	**Region**
2808	4.13	−27	−66	44	L inferior longitudinal fasciculus
2071	4.6	−11	−77	32	L inferior longitudinal fasciculus
749	2.98	−33	−83	24	L inferior fronto-occipital fasciculus
673	4.03	−29	−7	−8	L inferior fronto-occipital fasciculus
578	3.88	27	−39	17	R inferior fronto-occipital fasciculus
482	4.68	−33	−47	47	L superior longitudinal fasciculus
476	3.75	−44	−62	35	L superior longitudinal fasciculus

*The MNI coordinates and t-values for the local maxima of the centers of the clusters; R, right hemisphere; L, left hemisphere. The statistical threshold of all reported clusters were set at p < 0.05 using AlphaSim correction (with combination of threshold of p < 0.01 and a minimum cluster size of 473 voxels). The correlation analyses were adjusted for full-scale IQ, age and gender*.

**Table 2:** **Coefficient of line regression models**.

**Dependent variable**	**Predictors**	**Beta(Std.Err)**	**Beta'**	***t***	***p***
GM volumes	Age	−20.489(9.316)	−0.271	−2.199	0.032
	Sex	−4.310(7.336)	−0.071	−0.588	0.559
	IQ	0.014(0.431)	−0.004	−0.032	0.975
	Math scores	6.120(2.490)	0.350	2.457	0.017
FA values	Age	0.014(0.007)	0.236	2.093	0.043
	Sex	0.003(0.005)	0.069	0.601	0.551
	IQ	0.000(0.000)	−0.177	−1.324	0.193
	Math scores	0.011(0.002)	0.777	5.772	0.000

*Beta(Std.Err): Unstandardized coefficient (Standard Error)*.

*Beta': Standardized coefficient*.

## Discussion

The current study showed that structural properties of the brain, including GM volumes in the left IPS and FA values in the tract pathways adjacent to left IPS, were correlated with children's arithmetic performance. These correlations indicated that between-subject variability of human brain structure was associated with individual differences in schoolchildrens' arithmetic skills. First, we demonstrated that the arithmetic scores were positively correlated with GM volumes in the left IPS (Figures 1, 2). Second, voxel-based correlation analysis based on the fiber tracking results showed that FA values in the left SLF, ILF and bilateral IFOF (Figures 3, 4) were significantly and positively correlated with the arithmetic scores. The combination of the T1-weighted and DTI images provides further evidence that the macro- and micro-structures differences in the left IPS of schoolchildren are strongly associated with individual differences in arithmetic performance.

### Correlation between arithmetic scores and GM volumes

The parietal areas have long been implicated in numerical and mathematical processing in healthy individuals in both functional and structural imaging studies. For example, the anterior IPS is assumed to play a crucial role in cognitive representation of numerical quantities (Dehaene et al., 2003; Zhang et al., 2012). A recently published meta-analysis study showed that number and calculation tasks elicited activity in such region as the inferior parietal lobule (Arsalidou and Taylor, 2011). Children with greater activation in the left IPS during symbolic number processing exhibited higher arithmetic competence (Bugden et al., 2012). Recently, individual differences in functional activation were sometimes observed in regions exhibiting group differences in GM structure (Takeuchi et al., 2012). Furthermore, it is reported that a positive correlation between GM structure and certain cognitive functions is sometimes observed in regions where a positive correlation is observed between functional activation and certain cognitive functions. In the present study, the GM structure-behavior correlation we observed in the left IPS is possibly due to its engagement in numerical and mathematical processing (Bugden et al., 2012). The present finding confirmed the viewpoint that left IPS might underlie mathematical calculation.

In addition, we observed a positive correlation between arithmetic scores and the IPS GM volumes in the left hemisphere. This result is consistent with the previous finding that children with higher numerical proficiency have larger GM volumes in the inferior parietal regions including left IPS and bilateral angular gyri (Lubin et al., 2013). Brain structure in the left inferior parietal region seems to be consistently involved in mathematical tasks across both healthy (Aydin et al., 2007; Lubin et al., 2013) and abnormal subjects with arithmetic dysfunction (Rykhlevskaia et al., 2009; Lebel et al., 2010). It has been previously shown that the cortical GM density in the left inferior parietal lobule was significantly higher in mathematicians than in the control subjects (Aydin et al., 2007). Moreover, studies on children with neurogenetic disorders and cognitive disorders suggested that mathematical difficulties might be related to GM abnormalities in the inferior parietal regions and WM regions adjacent to the inferior parietal (Barnea-Goraly et al., 2005; Rykhlevskaia et al., 2009; Lebel et al., 2010). For example, dyscalculic children exhibited GM loss in the left IPS as compared to the control subjects (Rykhlevskaia et al., 2009). These results suggest that left IPS plays a consistent and important role in performing mathematical tasks. Our results are in line with the previously mentioned findings. The present study indicated that the structural differences in left IPS were associated with individual differences in schoolchildrens' math ability.

### Correlation between arithmetic score and WM FA

In the present study, the tractography analysis examined the fiber tracts running through the left IPS. Based on the JHU WM tractography atlas, we identified the ILF, SLF and IFOF as key fiber tracts running through this region. The voxel-wise regression analysis in the probabilistic pathways showed that schoolchildrens' FA values in the left SLF, ILF and bilateral IFOF are positively correlated with their arithmetic scores. FA reflects the directionality of water diffusion in the tissue, and is generally regarded as a measure of WM integrity (Johansen-Berg and Behrens, 2009). The brain-behavior correlations reported above indicated that children who were better at math would show higher WM integrity in these regions.

The SLF, which runs in an anterior-posterior direction, connects the parietal lobe association cortices with the frontal lobe, and travels through part of the WM of the superior frontal gyrus, terminating in dorsolateral and -medial frontal cortices (Schmahmann and Pandya, 2009). Previous research has shown that the SLF is actually composed of four separate components (Makris et al., 2005). According to this finding, the SLF in the present study is actually the anterior portion of the left SLF, which was found to connect inferior parietal and inferior frontal cortex (Tsang et al., 2009). Some recent studies (Tsang et al., 2009; Lebel et al., 2010; Matejko et al., 2013) suggested that the integrity of left-lateralized parietal WM structures was related to mathematical cognition. A positive correlation was observed between children's FA values in the anterior portion of the left SLF and their mental arithmetical performance (Tsang et al., 2009). These results indicated that FA in the anterior portion of the left SLF was related to number processing. Moreover, the study by Matejko et al. (2013) provided insights into the WM microstructures associated with arithmetic scores on the Preliminary Scholastic Aptitude Test. They found that FA values in the left parietal WM areas (specifically in the left SLF, superior corona radiata, and corticospinal tract) were positively correlated with arithmetic scores. Another study involving children with fetal alcohol spectrum disorder also reported that FA values in the left SLF were related to arithmetic scores (Lebel et al., 2010).

The ILF is commonly considered to be a long association fiber bundle interconnecting the occipital and temporal lobes (Catani et al., 2003). The IFOF is also found to connect the occipital and frontal lobes via the temporal lobe (Kier et al., 2004). As previously described, numerous publications have demonstrated that left ILF and IFOF might have a important role in the language semantic processing (Mandonnet et al., 2007; Martino et al., 2010). Studies on the brain mechanisms underlying mental arithmetic indicated that verbal processes were involved in calculation (Dehaene et al., 1999, 2003). So the brain-behavior correlation in ILF and IFOF in the present study demonstrated that children who were more successful in calculation were more efficient in the verbal-based processing. The structural regions, such as ILF and IFOF, were more involved in the calculation processing and a positive correlation between the FA values and the arithmetic scores was observed in these regions. Besides, the association between arithmetic scores and WM integrity in the ILF in the present study may be comparable to the results of the DTI study of schoolchildren by van Eimeren et al. (2008). Atlas-based tract mapping identified the ILF, IFOF and caudal forceps as key pathways impaired in dyscalculia children who are impaired in numerical competence and arithmetic skills (Rykhlevskaia et al., 2009). These evidences supported the observed associations between arithmetic scores and FA values in the present study. The relationship in the present study revealed that these WM microstructures were most strongly related to individual differences in arithmetic achievement.

### Brain development, learning and disorder

Brain development is a highly coordinated and sequenced event, involving both progressive and regressive processes. During the process of brain development, brain functions and the underlying neurostructures could be modulated by learning (Zatorre et al., 2012). Taken together, the results in the present study provided new evidence for the behavioral relevance of brain structural variation. Both schoolchildrens' GM volume and WM FA values were correlated with their arithmetic scores, which suggests that left IPS is a key neural substrate for representing children's arithmetic skills. In addition, we quantified and visualized the brain-behavior correlation by using a combination of T1-weighted images and DTI images. The current findings may offer great potential to further our understanding of individual differences in arithmetic achievement. Moreover, the structure-behavior relationship may improve our understanding of the structural plasticity of brain regions underlying arithmetic competence, which has vast clinical implications for treatments of brain disorders, including developmental dyscalculia and brain lesions. Appropriate learning experience is likely to serve as an intervention to improve or prevent those mental disorders.

The present study has some limitations. First, this study cannot completely eliminate the influence of brain maturation on the observed brain-behavior relationship, though we controlled the influence of the other factors. A study with a longitudinal design may provide a better understanding of this relationship and disentangle the different contributions of brain maturation and mathematical learning experience. Second, since previous findings indicated a link between functional activation and structural property (van Eimeren et al., 2010; Takeuchi et al., 2012), further studies delineating the complex relationships between brain function, structure and behavior are highly needed.

## Conclusion

In conclusion, we analyzed brain-behavior relationship using a combination of T1-weighted images and DTI data. The structural properties, including GM volumes in the left IPS and FA values in the left SLF, ILF and bilateral IFOF, were significantly and positively correlated with individual differences in arithmetic achievement. The present findings suggest that structural differences in children's intracortical GM region and the subcortical WM region are associated with individual differences of their arithmetic achievement. Importantly, the left inferior parietal regions, including the GM cortical regions and the adjacent fiber tracts, were identified as a key neural substrate for the anatomical correlation to arithmetic achievement. Finally, the results of correlation analysis provide insights into the brain structural correlates of cognitive function. These data may shed some light on understanding the structural correlates of math learning and treating developmental dyscalculia.

### Conflict of interest statement

The authors declare that the research was conducted in the absence of any commercial or financial relationships that could be construed as a potential conflict of interest.
